# Brainstem tumors may increase the impairment of behavioral emotional cognition in children

**DOI:** 10.1007/s11060-022-04161-x

**Published:** 2022-11-04

**Authors:** Heyuan Jia, Peng Zhang, Guocan Gu, Tian Li, Zhuang Jiang, Zhen Wu, Liang Wang, Junting Zhang, Yunyun Duan, Yaou Liu, Feng Yang, Shaozheng Qin, Liwei Zhang

**Affiliations:** 1 School of Instrumentation and Optoelectronic Engineering, Beihang University, No.37. BeiHang University XueYuan Road, HaiDian District, Beijing, 100083 China; 2 Beijing Advanced Innovation Center for Big Data-Based Precision Medicine, Beihang University, BeiHang University XueYuan Road, HaiDian District, BeiJing, 100083 China; 3 Department of Neurosurgery, Beijing Tiantan Hospital, Capital Medical University, No. 119. the West Southern 4Th Ring Road, Fengtai District, Beijing, 100073 China; 4 Department of Radiology, Beijing Tiantan Hospital, Capital Medical University, No. 119. the West Southern 4Th Ring Road, Fengtai District, Beijing, 100073 China; 5 China National Clinical Research Center for Neurological Diseases, Beijing Tiantan Hospital, Capital Medical University, No. 119. the West Southern 4Th Ring Road, Fengtai District, Beijing, 100073 China; 6 Advanced Innovation Center for Human Brain Protection, Capital Medical University, Beijing, 100070 China; 7 State Key Laboratory of Cognitive Neuroscience and Learning & IDG/McGovern Institute for Brain Research, Beijing Normal University, No. 19, XinJieKouWai St., HaiDian District, Beijing, 100875 China

**Keywords:** Brainstem tumors, Diffuse intrinsic pontine glioma, Behavioral problems, Emotional problems, Child behavior checklist

## Abstract

**Purpose:**

It remains unclear as to whether patients with brainstem tumor experience complex neuropsychiatric problems. In this cohort study, we specifically investigated behavioral, emotional and cognitive symptoms in pediatric patients with brainstem glioma and healthy individuals.

**Methods:**

A total of 146 patients with pediatric brainstem tumors (aged 4–18 years old) and 46 age-matched healthy children were recruited to assess their behaviors and emotions examined by the Child Behavior Checklist. A variety of clinical factors were also analyzed.

**Results:**

There were significant differences in most behavioral and emotional symptoms between pediatric patients and healthy subjects. Moreover, patients with pons tumors exhibited significantly higher scores than patients with medulla oblongata tumors (p = 0.012), particularly in concerning the syndrome categories of Withdrawn (p = 0.043), Anxious/depressed symptoms (p = 0.046), Thought Problems (p = 0.004), Attention deficits (p = 0.008), Externalizing problems (p = 0.013), and Aggressive behavior (p = 0.004). A tumor body located in the pontine (p = 0.01, OR = 4.5, 95% CI = 1.4–14.059) or DIPG in the midbrain (p = 0.002, OR = 3.818, 95% CI = 1.629–8.948) appears to act as a risk factor that is associated with more problems in patients with neuropsychiatric symptoms.

**Conclusions:**

Pediatric patients with brainstem tumors exhibit severe behavioral and emotional problems. Tumor invades the pontine and midbrain act a risk factor with more problems. It suggests that structural and functional abnormalities in the brainstem will cause prolonged behavioral problems and emotional-cognitive dysfunctions in young children.

**Supplementary Information:**

The online version contains supplementary material available at 10.1007/s11060-022-04161-x.

## Introduction

Pediatric brainstem tumor is a rare but fatal disease, which accounts for 10–20% of the tumors affecting the central nervous system in children [1]. The median age at the time of diagnosis has been recorded as young as 6 to 7 years old, and the disease equally affects males and females [2]. Among pediatric brainstem tumors, diffuse intrinsic pontine glioma (DIPG) is considered to be the most pervasive one. In fact, it represents an aggressive form of glioma that is characterized by the worst prognosis. It accounts for more than 80% of pediatric brainstem glioma cases [3]. The median survival for the patients with DIPG has been reported to be less than one year. Clinical manifestations of brainstem glioma mainly involve vertebral tract signs (such as limb weakness or paralysis), posterior nerve dysfunction, and cerebellar disturbances, which are primarily associated with tumor location and growth pattern. Patients present these symptoms either individually or in combination with rapid onset and short duration (1–2 months) [4].

To date, few studies have explored the role of the brainstem in cognitive and emotional functions [5]. Evidence from Keschner, however, has pointed toward “mental/psychological symptoms” in patients with brainstem tumors [6].Since then, there has been an increase in the reported number of cases with behavioral, cognitive, and affective dysfunctions following a diagnosis of brainstem tumors. Children with tumors in the brainstem had been reported with irritability, hostility, lack of cooperation, attention deficit, stereotyped behavior, woke up at night, pathological smile and cry. And adult patients had been reported with anxiety, paranoid, forgetfulness, loss of interest, dyscalculia, executive function and the decrease of the general intelligence [7–11]. In addition to tumors, a neuropsychological study had found that a small infarct in the brainstem may affect cognitive function in a nonspecific way [12]. The cognitive, emotional, and behavioral dysfunctions caused by independent brainstem lesions are not specific. Probably because the brainstem is and inherent part of the cerebellar cognitive network. The damage of brainstem may cause a range of symptoms similar to cerebellar cognitive affective network (CCAS) [11, 13, 14]. Moreover, neurotransmitter neurons which concentrated in the brainstem can regulate the activity of supratentorial brain regions [15]. Therefore, the brainstem regulates behavior, cognition, and emotion through close connections with cerebellum and cerebral cortex.

However, “psychological and cognitive deficits” still receive less attention by most neurosurgeons. Despite the clinical importance, little systematic research has been conducted in this field. Unfortunately, parents tend to downplay abnormal behaviors in children caused by tumors, until the development of obvious physical symptoms. Potential abnormal behaviors and deficits in cognitive and emotional functions can be evaluated using neuropsychological tools. Currently, the Child Behavior Checklist (CBCL) is a parent‐based rating scale, which is most widely utilized for the assessment of children’s behavioral and emotional problems [16]. However, direct evidence is still lacking about whether and how children with brainstem tumors exhibit abnormal behaviors and emotional problems.

To address these questions, we set up the present study to investigate abnormalities of behavioral and emotional functions in children with brainstem tumors. The CBCL was used to assess children’s behavioral and emotional problems. Then we compared results with healthy children. Group differences in behavioral and emotional symptoms were examined between patient and control groups. Correlations of these outcomes with pathological and radiographical features of brainstem tumors were also examined in the patient group.

## Materials and methods

### Participants

**Fig. 1:**
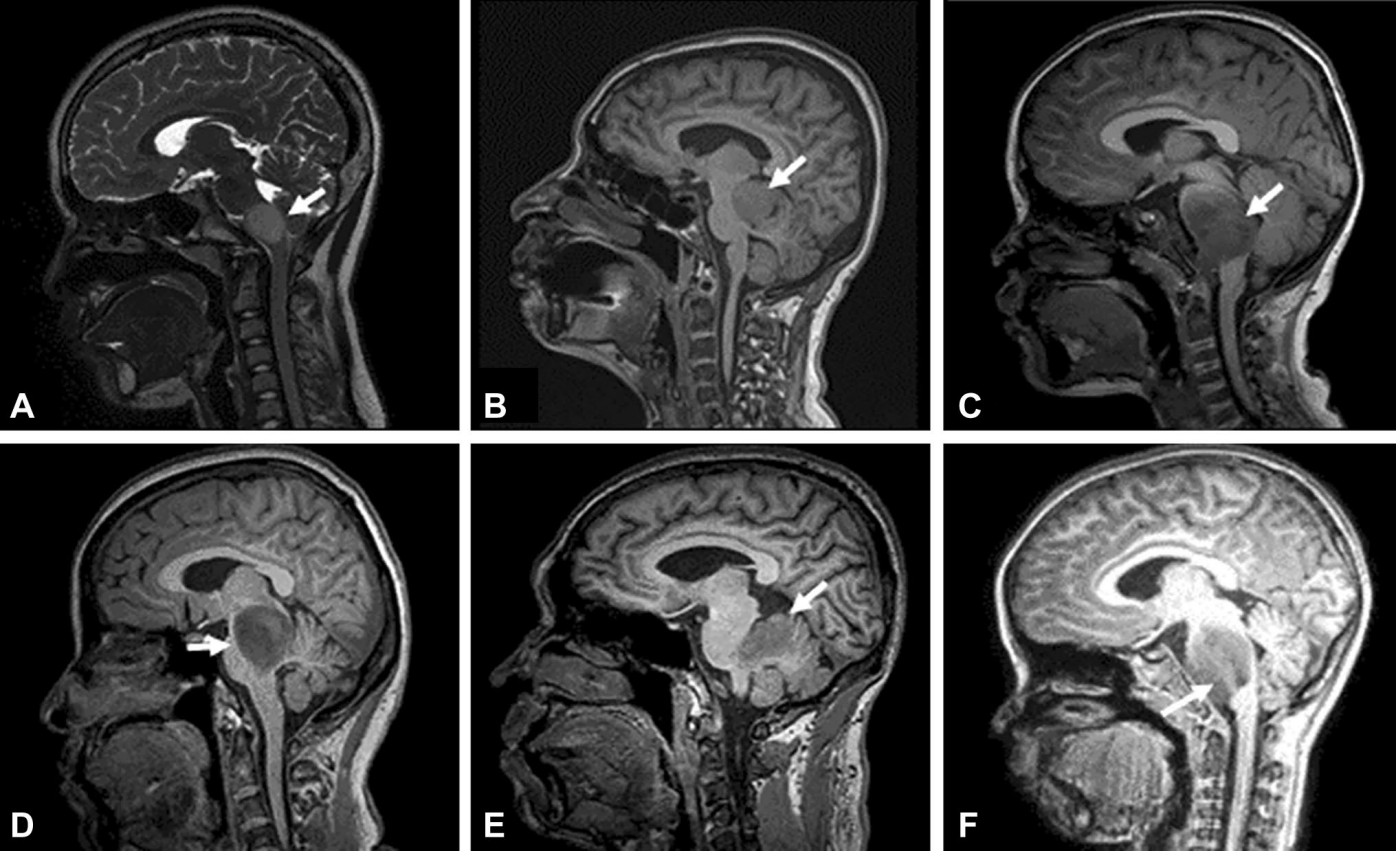
Brain MRI of children with a brainstem tumor. **A** T2-weighted Sagittal FLAIR MRI image showing a tumor located in the medulla oblongata. **B** T1-weighted Sagittal FLAIR MRI image showing a tumor located in the midbrain. **C** and a tumor located in pons **D** DIPG extending to the midbrain. **E** DIPG extending to the cerebellum. **F** DIPG extending to the medulla oblongata. The white arrows point to the tumor body

We collected the medical data of 146 patients (age: 4–18 years) that were emitted from April 2019 to March 2022 with a diagnosis of brainstem tumors to the Department of Neurosurgery, Beijing Tiantan Hospital, Capital Medical University. All of these patients were enrolled in our study. The magnetic resonance image (MRI) features of brainstem tumors are shown in Fig. 1. The exclusion criteria for selection of the subjects included the following: (a) history of tumor treatment; (b) inadequate imaging examination; (c) diagnosis of mental illness; (d) any previous trauma, neurological disorders, or severe physical disease. A total of 46 local-residing healthy children with no physical and mental illnesses, who were matched for sex, age, and education, were recruited as controls. Informed consent was provided by the parents of all participants as per the Institutional Review Board-approved protocol obtained from the Beijing Tiantan Hospital, Capital Medical University. All participants' parents completed the CBCL before any further examination and treatment in their children. The population and clinical characteristics, such as age, sex, years of education, and medical history, were collected during the interview. The pathology and imaging metrics of tumors (e.g., location, affected areas, the presence of transverse fiber in pontine) were obtained from the patients’ medical records.

### Outcome measures

The CBCL scale is currently the most widely used parent observation-based form for describing behavioral and emotional problems in school-age children (suitable for 4–18-year-old). The whole scale contains capability entries and problem areas. The former is used to evaluate children’s activity, social relations, and learning abilities. The latter includes 113 items that can be classified into 8 syndromes including Withdrawn, Somatic Complaints, Anxious/Depressed, Social Problems, Thought Problems, Attention Problems, Delinquent Behavior, and Aggressive Behavior. Among these, Withdrawn, Somatic Complaints and Anxious/Depressed were summarized as Internalizing Problems. Delinquent Behavior and Aggressive Behavior were categorized as Externalizing Problems [17]. The Chinese version of CBCL was designed for improvements by the Shanghai Mental Health Center and has been validated to show good reliability and validity [18]. This scale requires parents to rate the frequency of children’s behavior in the last 6 months (0 = 'no such performance', 1 = 'occasional performance', 2 = 'frequent or significant performance'). It takes approximately 15 min to complete the entire scale. The higher scores on capability items equal to better activity, social condition and school condition in children. In contrast, higher scores of problem items indicate more behavioral-emotional problems. Scores that above the 98^th^ percentile of the norm are in the clinical range [17]. According to Liu’s suggestion [19, 20], the cut-off value of Total Problem score for Chinese children is 35 points.

### Statistical analysis

All analyses for descriptive statistics were performed with the SPSS (Version 26.0). The scores of Total Problems, Internalizing Problems, Externalizing Problems, and Eight Syndromes were calculated. To characterize behavioral and emotional symptoms in patients and healthy children, we employed descriptive statistics (i.e., means, standard deviations (SDs), medians, frequencies, and average rankings). We also assessed the prevalence of all problems in different cohorts (patients vs. healthy children, boys vs. girls, young cohort aged 4-11y vs. old cohort aged 12-18y). Not all variables followed a normal distribution. As a result, the Mann–Whitney U-test was applied to compare the subgroup differences. The main and interaction effects of the subgroups were studied by analysis of covariance (ANCOVA) To identify the independent risk factors for abnormal behavior in patients whose total problems exceeded 35 points, we applied the multivariate logistic regression analyses to analyze all significant factors in the univariate analysis. The results are shown as odds ratio (OR), 95% confidence intervals (CIs), and p-values. Two-tailed statistical tests with 0.05 as the significant level cutoff were used to determine the statistical significance.

## Results

### Participant demographics and cohort features

**Fig. 2:**
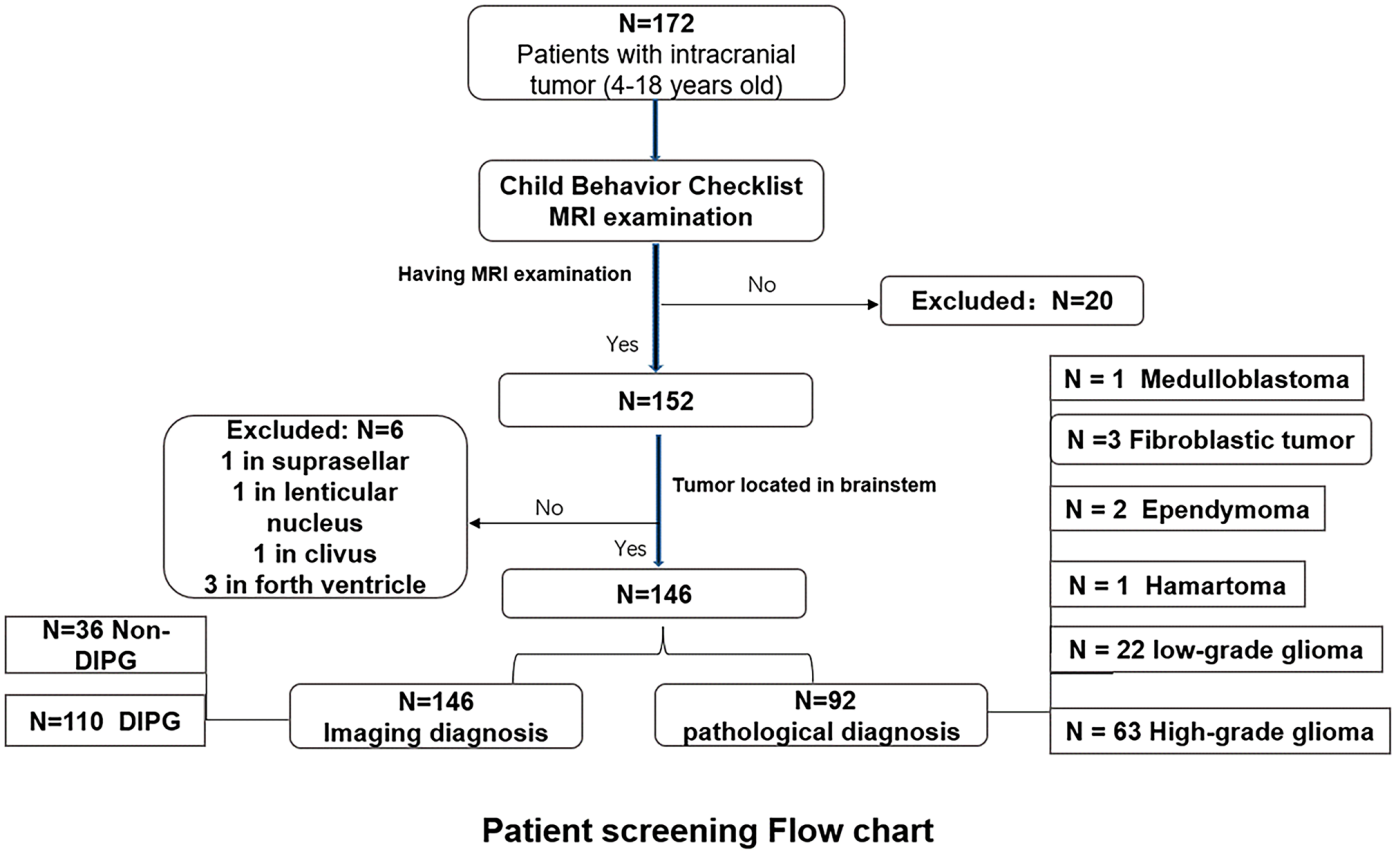
Patient screening flow chart

The present study initially involved a total of 172 patients with brainstem tumors and 46 local-residing healthy children, who were invited to fill out the CBCL. Among these, 20 patients without having a further MRI examination at the hospital and six patients without any tumor bodies discovered in the brainstem were excluded from the study. The final sample sizes consisted of 146 children with a confirmed diagnosis of brainstem tumors and 46 healthy children (Fig. 2). No significant differences were found between patients and healthy children, in terms of age (*t *_*(190)*_ = 0.822, *p* = 0.412), sex (χ^2^_*(1)*_ = 0.154, *p* = 0.736), or educational level (*t *_*(190)*_ = 1.41, *p* = 0.158).

Table 1 summarizes the characteristics of the research subjects included in this study. Overall, the average age of the patients was recorded to be 7.9 (SD 3.34) years, at the time of the interview. Among these, 55.5% of the patients were male. The average educational level was documented to be 2.52 (SD 2.82) years. Importantly, 63.01% of the patients had been subjected to surgical treatment or biopsy after approximately one month in Tiantan Hospital. In the patient group, 85 patients exhibited biopsy‐proven gliomas (63 high‐grade and 22 low‐grade, respectively), while three patients were pathologically diagnosed with a fibroblastic tumor, two with ependymoma, one with Medulloblastoma, and one with hamartoma after surgical treatment. For the remaining 54 patients, the diagnosis was made based on MRI findings. DIPG with the distinctive imaging features was categorized by two neurosurgeons according to radiological criteria of being derived from the pontine, presence of diffused growth pattern, and invasion of ≥ 50% of the pontine [21–23]. In the present study, 110 patients were diagnosed with DIPG.

**Table 1:** Cohort demographics and clinical data for patients with tumors and healthy children

Variables	Patients n (%)	Healthy children n (%)	P-value
Sex	146	46	0.695
Male	81(55.5%)	24(52.2%)	
Female	65(44.5%)	22(47.8%)	
Age, mean (SD), y	7.90(3.34)	8.37(3.43)	0.412
Younger	122(83.5%)	35(76.1%)	
Older	24(16.5%)	11(23.9%)	
Education level, mean (SD), y	2.52(2.82)	3.22(3.18)	0.158
Tumor type			
Pathological diagnosis (n = 92)			
Glioma	85		
High-grade	63		
Low-grade	22		
Fibroblastic tumor	3		
Ependymoma	2		
Hamartoma	1		
Medulloblastoma	1		
Imaging diagnosis (n = 146)			
DIPG	110		
Non-DIPG	36		

*DIPG* diffuse intrinsic pontine glioma

### Behavioral problems and emotional-cognitive dysfunctions in children with brainstem tumors

The rank means of CBCL scores for categories of Total Problems, Internalizing Problems, Externalizing Problems, and eight syndromes in patients and healthy children are shown in Table 2. When compared with healthy children, patients scored significantly higher in terms of Total Problems *(z* = *−*4.86*, p* < 0.0001), Internalizing Problems (*z* = *−*5.06*, p* < 0.001), Externalizing Problems (*z* = *−*2.42*, p* < 0.015), and among all of the other seven listed syndromes (*z* < *−*2.4*, p* < 0.05), with exception of Delinquent Behavior (*z* = *−*1.665*, p* = 0.096). Most markedly, patients scored significantly lower in those of Activity (*z* = *−*2.97*, p* = 0.003) and Social Condition (*z* = *−*2.61*, p* = 0.012). Although there were gender and age differences in some behavioral problems, the results of ANCOVA showed that brainstem lesions had more significant impact on behavioral problems.

**Table 2:** Data for different cohorts based on behavioral and emotional problems

CBCL	Tumor			Age group			Gender			ANCOVA		
	HC	Patients	p	Younger	Older	p	Boys	Girls	p	Tumor	Age	Gender
Total, n	46	146		157	35		105	87				
Activity	117.59	89.86	0.003**	98.94	85.57	0.206	94.34	99.11	0.210	0.003**	0.378	0.294
Social condition	114.29	90.89	0.012*	92.11	116.21	0.020*	91.95	101.99	0.468	0.007**	0.955	0.236
School function	98.92	95.74	0.732	92.88	112.76	0.054	99.14	93.31	0.018*	0.525	0.001**	0.083
Withdrawn	69.83	104.90	0.000***	97.36	92.63	0.645	87.92	106.86	0.722	0.000***	0.639	0.276
Somatic complaints	49.34	111.36	0.000***	96.72	95.53	0.908	88.61	106.02	0.029*	0.000***	0.128	0.002*
Anxious/depressed	75.05	103.12	0.003**	98.68	86.71	0.242	95.88	97.25	0.862	0.003**	0.632	0.491
Social problems	58.86	108.36	0.000***	100.54	78.40	0.031*	99.50	92.88	0.406	0.000**	0.641	0.499
Thought problem	79.53	101.85	0.012*	98.54	87.34	0.252	97.98	94.72	0.667	0.011*	0.734	0.717
Attention Problem	63.09	107.03	0.000***	97.83	90.51	0.479	103.28	88.32	0.062	0.000*	0.344	0.087
Delinquent behavior	84.91	100.15	0.096	99.66	82.34	0.087	100.60	91.56	0.241	0.069	0.352	0.324
Aggressive behavior	79.36	101.90	0.016*	100.97	76.46	0.018*	100.35	91.85	0.297	0.009**	0.749	0.687
Total problem	61.78	107.44	0.000***	99.39	82.01	0.088	98.08	94.59	0.671	0.000**	0.338	0.786
Internalizing	60.40	107.87	0.000***	97.39	92.49	0.636	93.09	100.63	0.351	0.000**	0.357	0.071
Externalizing	78.23	101.94	0.015*	100.87	76.91	0.021*	100.68	91.46	0.258	0.010*	0.636	0.579

Younger, < 12 years old; older, ≥ 12 years old

*CBCL* achenbach child behavior checklist, *HC* healthy children, *ANCOVA* analysis of covariance

*p < 0.05, **p < 0.01, ***p < 0.001

In the patient group, eleven patients exhibited tumor in the midbrain, 124 in the pontine, and eleven in the medulla. Following pairwise comparisons, patients with tumors located in the midbrain and pons were found to exhibit higher scores as compared to patients with tumors in the medulla. Especially in following categories: Withdrawn (*z* = 6.287*, p* = 0.043), Anxious/Depressed (*z* = 6.166*, p* = 0.046), Thought Problems (*z* = 11.196*, p* = 0.004), Attention Problems (*z* = 9.597*, p* = 0.008), Externalizing Problems (*z* = *−*2.421*, p* = 0.013), Aggressive Problems (*z* = 10.953*, p* = 0.004), and Total Problems (*z* = 8.93*, p* = 0.012) (See Supplementary Fig. 1A). Patients with tumor entities crossing the midline scored higher in those of Withdrawn (*z* = *−*2.172*, p* = 0.03), and Social Problem (*z* = *− *1.971*, p* = 0.049) (See Supplementary Fig. 1B). Patients diagnosed with DIPG showed higher scores in that for Aggressive Behavior (*z* = *−*2.184*, p* = 0.029), and Externalizing Problems (*z* = *−*1.984*, p* = 0.047) (See Supplementary Fig. 1C). Next, we assessed the involvement of adjacent brain regions in patients diagnosed with DIPG. The results were exhibited in Supplementary Fig. 2.

Furthermore, we analyzed the differences within the patient group in terms of gender and age. The results showed that male patients scored lower than female patients in terms of the School Condition (z = *−*2.475*, p* = 0.013) and Somatic Problem (*z* = *−*2.952*, p* = 0.003) categories. Additionally, older patients exhibited higher scores in those of Social Condition (z = *−*2.53,* p* = 0.011), School condition (*z* = *−2.611, p* = 0.009); whereas, younger patients scored higher in Delinquent Behavior (*z* = *−*2.108*, p* = 0.035), Aggressive Behavior (*z* = *−*2.19*, p* = 0.029), and Externalizing Problems (*z* = *−*2.21*, p* = 0.027) (See Supplementary Fig. 3).

Finally, confounding factors were considered, and we identified several major risk factors responsible for more problems in patients by implementing binary logistic regression analysis. The location of the tumor body remained simultaneously significant in the final multivariate model (*p* < 0.001). Importantly, the occurrence of tumor body in the in the pontine (*OR *4.5,* 95% CI *1.4–14.059*, p* = 0.01) or patients with DIPG involving the midbrain (*OR *3.818*, 95% CI *1.629*–*8.948,* p* = 0.002) acted as risk factors that were associated with more overall problems in the patients (Table 3).

**Table 3:** Clinical risk factors for behavioral and emotional problems of patients with brainstem tumor

Variables	p	OR	95% CI
Age	0.112		
Gender	0.743		
DIPG	0.982		
Involving midline	0.140		
Location			
Pontine midbrain & medulla	0.010*	4.5	1.4–14.059
Effected areas in DIPG			
Midbrain	0.002**	3.818	1.629–8.948
Pontibrachium	0.876		
Medulla	0.364		
Cerebellum	0.565		

*OR* odds ratio, *CI* confidence intervals

*p < 0.05, **p<0.01, ***p < 0.001

## Discussion

The present study examined behavioral and emotional problems and cognitive dysfunctions in 146 children with brainstem tumors that were not subjected to any previous treatment as compared with 46 age-matched healthy children. Our study showed that patients suffering from brainstem tumors performed worse in terms of activity, school function, and social function, and they presented more severe behavioral and emotional problems, when compared to healthy controls. The location of the tumor body within the brainstem significantly affected the results. Those with tumor body in pontine and midbrain have more behavioral problems than in medulla. Noteworthily, children diagnosed with DIPG showed more aggressive and externalized behaviors than others.

Several previous studies have suggested the involvement of the brainstem in several cognitive and emotional functions [13]. In fact, various cortical and subcortical regions are known to be involved in perception, appraisal and regulation of emotions. Among these, brainstem nuclei located in the pontine and midbrain tegmentum have been thought as the most important loci in modulating behavior, cognition and emotion [24]. Therefore, the term “emotional brainstem” was proposed to describe the brainstem’s role in emotion [25]. Likewise, brainstem nuclei were subdivided into three networks based on their role in emotion generation, activation and regulation. Hence, it is conceivable that disturbances in the "emotional brainstem" pathway might lead to abnormal behavioral and emotional functioning in patients with tumors. Since the brainstem serves as the source of norepinephrine, dopamine, and serotine, it modulates cortical circuits subserving emotion, cognition, and behavior. Most importantly, the locus coeruleus is known to play a key role in emotional functioning, primarily owing to the occurrence of the center of noradrenergic projections [15].

Previous study found that some children diagnosed with DIPG had deficit in behavioral inhibition [26]. Our study not only reached similar results, but also extracted potential influencing clinical factors. Furthermore, we observed that the location of the tumor body affected children’s behavioral and emotional problems independently. Specifically, more behavioral problems especially thought problems, attention problems, and aggressive behavior were found in patients with tumors in the pontine and midbrain in our studies. Accumulating evidence highlights the role of brainstem reticular formation in sleep, wakefulness, and alertness [27]. The neural circuit centered on the locus coeruleus is particularly important for selected attention [28, 29]. Previous studies, however, demonstrated that neither the pontine nor midbrain exhibited any direct involvement in cognition based on brainstem lesions induced by hypometabolism or neurochemical changes [12]. Recent studies suggests that there are deficits in behavioral and cognitive functions in patients with brainstem tumors, which might be attributed to the interruption of projections from the cortex to the brainstem. In vivo animal studies identified a fronto‐pontine loop [30] and cerebro‐cerebellar circuit, which included afferent cortico‐ponto‐cerebellar pathways and efferent cerebello‐thalamic‐cortical pathway [14].

Notably, patients with tumors located in the midbrain and pontin exhibited more aggressive behaviors in the present study. Conventionally, previously accumulated neuroscientific evidence suggested that the amygdala, hypothalamus, and periaqueductal (PAG) matter make up the subcortical circuits that are involved in aggression [31]. Additionally, it has been reported that the PAG region receives input of aggression from ventrolateral areas of the ventromedial hypothalamus [32]. Recently, it was hypothesized that the hypothalamic‐midbrain circuit represented organized social signals in aggressive behaviors. Thus, the inactivation of PAG cells resulted in aggression‐specific deficits [33]. However, in the present study, we found that patients with glioma and damaged midbrain (mostly involving the PAG) showed higher levels of aggression. The brainstem participates in physiological mechanisms of aggression, such as through the serotonin and norepinephrine systems. In addition to this, emotional and cognitive dissonance might cause aggressive behavior [34]. Interestingly, the role of the pontine in aggression has been reported before in only a few studies [35].

DIPG patients with midbrain lesions might lead to the development of social interaction problems. Consistent with this, patients in our study with tumors extended to the cerebellum exhibited worse social function. In general, social interaction problems are closely related to social attention skills, which has been previously shown to involve midbrain and limbic‐level functioning [36]. A prospective auditory brainstem response study demonstrated that infants born with brainstem dysfunction developed social attention deficit and social avoidance disorders in childhood [37].

### Clinical implications

Brainstem gliomas occurring in children undoubtedly present huge adverse events for their families. According to clinical observation, obvious physical symptoms occur in most of these children, roughly about 10 days to six months before primary diagnosis [4, 38]. Importantly, behavioral and emotional problems often occur much earlier. Unfortunately, parents tend to ignore this sudden onset of abnormal functioning, owing to their focus on dealing with more vital complaints. In fact, these manifestations are likely attributed by the parents to the child being "naughty". Our study confirmed that the CBCL could be utilized by parents and pediatrician to evaluate children’s behavior and mood. An advance in understanding emotional and cognitive abnormalities caused by brainstem lesions will aid the early detection of brainstem injury and the improvement of patients’ clinical care. Notably, co‐occurrence of high attention problems, aggressive behavior, and depressed/anxious problems in children has been shown to increase the risk of bipolar disorder (BD) development later in adult hood [39, 40]. A combination of internalizing and externalizing problems is indicative of self‐regulatory problems in children [39], which might further lead to more difficulty in dealing with life setbacks. In particular, such children present with lower activity, withdrawal, depression, and delinquent behaviors.

The present study also provides new insights into the functioning of the brainstem. That is, the brainstem not only contributes to basic life‐support functions (e.g., autonomous breathing), but it also plays an important role in emotional and behavioral regulation. The brainstem is necessary for the cerebellar‐cortical loops. Thus, damage to the brainstem might also lead to a series of symptoms similar to the cerebral cortical cognitive and affective syndrome [13]. Due to the small size of the brainstem, it is hard to separate its functions. The methods used in this study would contribute to the study of physiological and pathological mechanisms in separate regions of brainstem. For example, the present research studied the location and pathological characteristics of tumors. Results indicated that aggressive tumors (referred to DIPG) increase aggressive behaviors in children. Different lesion locations might cause diverse abnormal behaviors. In addition to this, our study may help facilitate neuropsychological advances that lead to establishing a model of impaired cognition for diagnosing brainstem tumors.

Previous studies suffered from the limitations of small sample sizes or the absence of clinical characteristic analyses. The present study offered certain advantages. In particular, this study used a scale for objective assessment of a range of behavioral and emotional problems in patients with brainstem tumors. Additionally, the study involved a large sample size and matched healthy children as a control group. The inclusion of clinical and imaging factors also acted as a strength of this study.

### Limitations

The present study has several limitations. First, the CBCL was used to identify behavioral and emotional problems rather than a clinical diagnosis. Thus, the classification of specific behavioral and emotional problems is only to be useful towards brainstem tumor diagnosis. Second, the current findings were derived from a structured cross‐sectional and retrospective self‐assessment questionnaire. Consequently, recall bias cannot be ruled out completely. Third, some other risk factors in early childhood, such as poor family relationships and low socioeconomic status, might also exert some effects [19]. These risk factors do not necessarily reflect causality in our study. Finally, as a brainstem injury can cause complex physical disorders, our study cannot predict whether patients’ abnormal behavior and mood are primary or secondary.

## Conclusions

Altogether, the present study demonstrates that children with brainstem tumors present some behavioral and emotional problems. Abnormal behaviors were found to exhibit an association with the location of the tumor body. In particular, patients with tumors located in the pons exhibited more behavioral problems as compared to those involving the midbrain medulla. No differences were discovered in patients’ behavior and emotional performance in terms of the pathological type of tumor. The CBCL was easy to implement to assess children’s abnormal behavioral problems, which might contribute towards the early detection of comorbid mental illness in youths with brainstem tumors. Understanding the role of the brainstem in neuro‐cognition would assist in the clinical diagnosis, improvement of clinical care, and timely application of effective treatment strategies to ensure brain protection.

## Supplementary Information

Below is the link to the electronic supplementary material.

Supplementary file1 (PDF 374 kb)

## Data Availability

The datasets generated during the current study are available from the corresponding author on reasonable request.
