# Supplementary figures and images for: Brainstem tumors may increase the impairment of behavioral emotional cognition in children
Source: J Neurooncol. 2022 Nov 4;160(2):423–32. doi: 10.1007/s11060-022-04161-x (PMC9722802; doi:10.1007/s11060-022-04161-x)

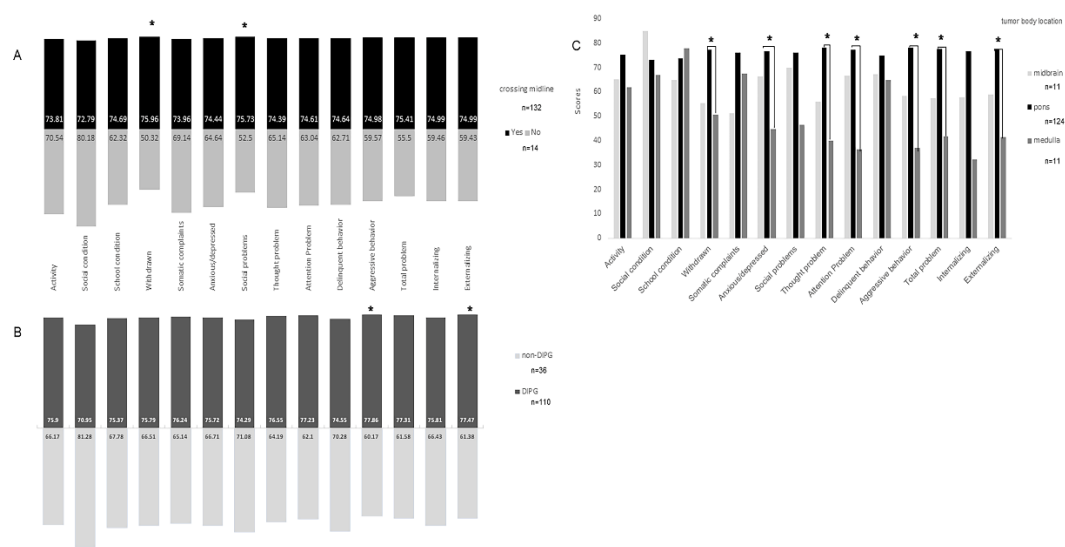

Supplementary fig 1

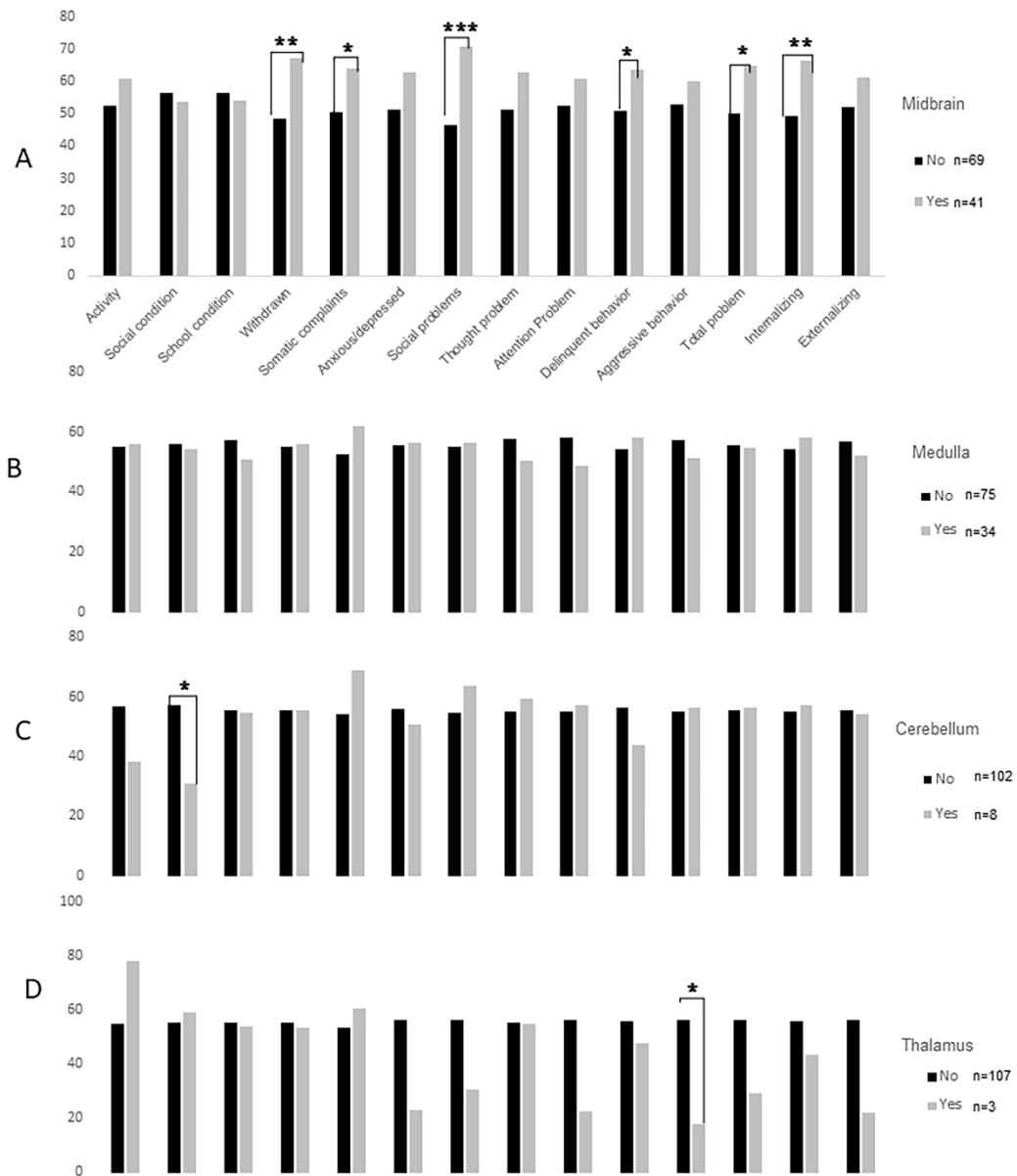

Supplementary fig 2

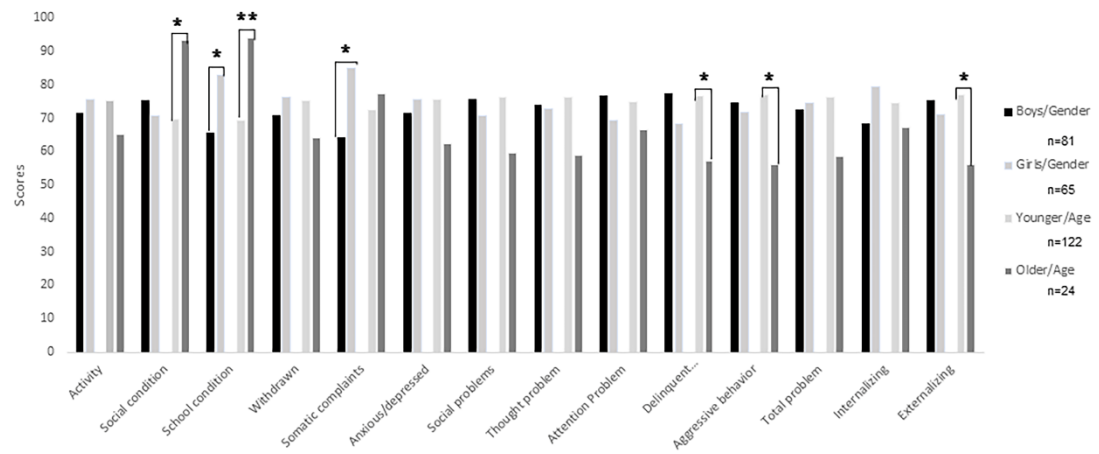

Supplementary fig 3

Supplement: Supplementary file 1 — Supplementary file1 (PDF 374 kb) [file 11060_2022_4161_MOESM1_ESM.pdf]
